# The Prevalence of Asymptomatic Bacteriuria in Iranian Pregnant Women: A Systematic Review and Meta-Analysis

**DOI:** 10.1371/journal.pone.0158031

**Published:** 2016-06-23

**Authors:** Mahin Ghafari, Vali Baigi, Zahra Cheraghi, Amin Doosti-Irani

**Affiliations:** 1 Department of Public Health, School of Health, Shahrekord University of Medical Sciences, Shahrekord, Iran; 2 Department of Epidemiology and Biostatistics, School of Public Health, Tehran University of Medical Sciences, Tehran, Iran; University of Missouri, UNITED STATES

## Abstract

**Background:**

Asymptomatic bacteriuria (ASB) is defined as the presence of bacteria in urine without having signs and symptoms. The aim of this meta-analysis was to estimate the overall prevalence of asymptomatic bacteriuria among Iranian pregnant women.

**Methods:**

Major national and international databases were searched up to November 2015, including Scientific Information Database, MagIran, Web of Science, Medline, Scopus, Science Direct and Ovid. The checklist of the STROBE statement was used for evaluating the quality of reporting. The extracted data were analyzed and the results were reported using a random-effects model with 95% confidence interval (CI).

**Results:**

From 3709 obtained studies, 20 included in the meta-analysis, which involved 15108 pregnant women. The overall prevalence of ASB was 0.13 (95% CI: 0.09, 0.17). The prevalence of ASB in the northern and southern regions of Iran was 0.13 (95% CI: 0.09, 0.18) and 0.11 (95% CI: 0.05, 0.16), respectively.

**Conclusion:**

Prevalence of ASB among Iranian pregnant women is considerable. Due to the complications of ASB for pregnant women and their children, preventative planning and control of ASB among pregnant women in Iran is necessary.

## Introduction

Asymptomatic bacteriuria (ASB) is defined as the presence of bacteria in urine without having signs and symptoms. This condition affects all groups, but women, particularly pregnant women, are more susceptible than men because of a short urethra and easy contamination of the tract with fecal flora [1] Asymptomatic bacteriuria (ASB) is defined as two consecutive voided urine specimens or one properly collected specimen of urine from pregnant women any signs and symptoms of urinary tract infection with isolation of the same bacterial strain in quantitative counts of 10^5^ cfu/mL. [2, 3].

ASB during pregnancy relates to the physiologic and anatomic changes in the urinary tract. The prevalence of ABS in pregnant women is estimated to be approximately 1.9–15% [4]. Pregnant women with ASB are at an increased risk for severe outcomes so that without antibiotic therapy, approximately 30% of pregnant women affected by symptomatic bacteriuria may have complications such as preterm delivery and low birth weight infants. In addition, the risk of developing pyelonephritis during pregnancy is approximately 20–30 fold higher than that in women without bacteriuria [5, 6]. Treatment of asymptomatic bacteriuria during pregnancy decreases the risk of subsequent complications [7]. Therefore, screening of pregnant women is necessary for early diagnosis and treatment of ASB and subsequent prevention of its complications [8]. Until now, several studies estimated the prevalence of asymptomatic bacteriuria among pregnant women in different regions of Iran [9–14]. However, there is controversy in the results of the conducted studies. The aim of this meta-analysis was to estimate the overall prevalence of asymptomatic bacteriuria among Iranian pregnant women.

## Methods

### Searching

This meta-analysis was performed according to PRISMA guideline [15]. Major national and international databases were used to search for the following key words: asymptomatic bacteriuria, pregnant women, prevalence, incidence, and Iran. The national databases that were used included the Science Information Database (up to November 2015) and MagIran (up to November 2015). The international databases included the Web of Science (January 1945–November 2015), Medline (January 1950–November 2015), Scopus (January 1973–November 2015), ScienceDirect (January 1823–November 2015) and Ovid (January 1860–November 2015).

The reference lists of included studies were scanned in order to obtain additional articles. The corresponding authors of the included studies were contacted as well.

### Criteria for including studies

All cross-sectional studies, which investigated the prevalence of asymptomatic bacteriuria in pregnant women in Iran, irrespective of language and date of publication, were retrieved. Iranian pregnant women living in Iran were considered as the study population. The main outcome of interest was the prevalence of asymptomatic bacteriuria. The one urine culture for diagnosis of ASB was acceptable for included the cross-sectional studies in this systematic review.

### Data collection and validity assessment

Two authors (VB and ADI) independently screened the title and abstract of retrieved articles and reviewed the full texts of the selected studies according to the inclusion criteria for this meta-analysis. Any disagreement between the two authors was resolved by verdict of a third author (ZC). The interested variables, which were extracted for data analysis included the year and location of study conduction, sample size, number of pregnant women with asymptomatic bacteriuria, type of diagnostic test, and type of bacteriuria.

Six selected items from Strengthening the Reporting of Observational studies in Epidemiology (STROBE) statement [16] were used for evaluating the quality of reporting. The items included (a) the eligibility criteria for including participants; (b) a clear definition of outcome, i.e., asymptomatic bacteriuria; (c) description of locations, settings, and relevant dates of studies; (d) demographic characteristics of pregnant women; (e) how the sample size was arrived; and (f) a report of the number of interested outcomes. The studies that satisfied all mentioned criteria were classified as high quality, studies that did not meet two criteria were classified as intermediate, and studies that did not meet more than two criteria were classified as low quality.

### Heterogeneity and statistical analysis

The statistical heterogeneity was explored using the chi-square (Chi2) test at 10% significant level. In addition, the heterogeneity across the results of the included studies were quantified by I2 statistic, and the between study variance was estimated using tau-square (Tau2) statistic [17, 18]. The statistical software Stata 11 (Stata Corp, College Station, TX, USA) was used for data analysis. Meta-analysis was conducted to obtain the overall prevalence of asymptomatic bacteriuria in pregnant women. The extracted data were analyzed and the results were reported using a random-effects model [19] with 95% confidence interval (CI). Sub group analysis was performed based on quality of included studies, geographic region of Iran, and year of study conduction.

## Results

### Description of included studies

**Fig 1:**
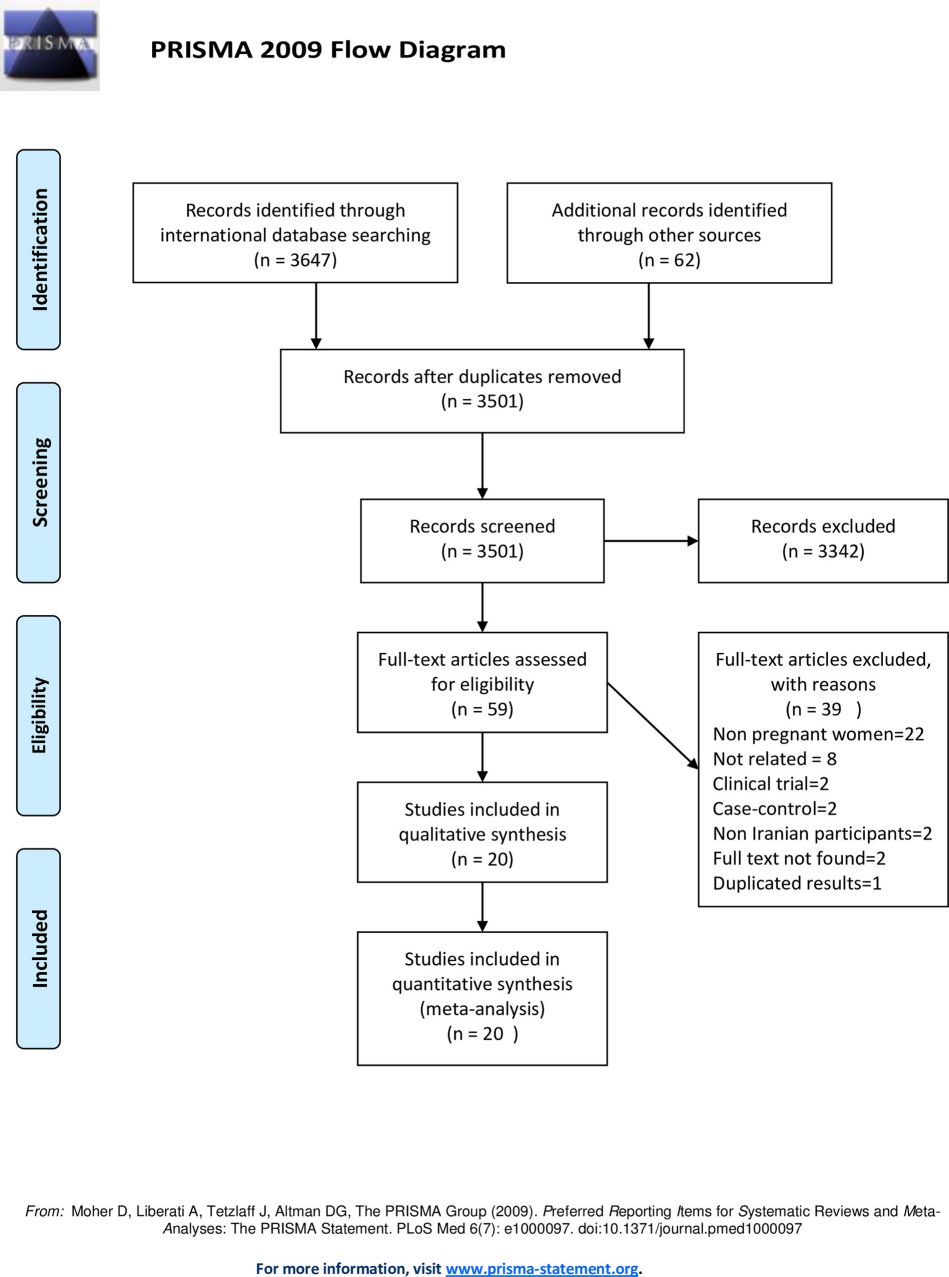
A flow chart depicting the stages of retrieving articles and checking eligibility criteria for meta-analysis.

We obtained 3709 studies up to November 2015 including 3647 studies from international database and 62 from national databases. Of the retrieved studies, 208 were excluded because of duplication, 3442 did not relate to the aim of this meta-analysis, 39 did not meet eligibility criteria, and 20 were included in the meta-analysis [9, 10, 12–14, 20–34] that involved 15108 pregnant women (Table 1 and Fig 1).

**Table 1:** Characteristic of included studies in meta-analysis.

Study	City	Mean age	Sample size	Number of patient	Type of bacteria	Prevalence of ASB	Test	Criteria for diagnostic (NB in ml)
Azhari., 2012	Sabzevar		1100	39	E.coli	0.04	Culture	NR
Dadkhah, 2010	Tehran (Akbarabadi Hospital)		1246	113	NR	0.09	Urinalysis and culture	10^5
Danesh, 2009	Isfahan		172	42	E.coli	0.24	Urinalysis and culture	N.R
Daneshyar, 2010	Hamadan		377	38	Staphylococcus epidermidis	0.10	Culture	10^5
Farajzadegan, 2008	Isfahan	25.3	200	8	NR	0.04	Urinalysis and culture	10^5
Ghafarnejad, 1998	Tehran(Mirzakuchackhan Hospital)		205	14	NR	0.07	Urinalysis and culture	10^5
Hazhir, 2007	East Azerbaijan		1100	647	NR	0.59	Culture	N.R
Kalantar, 2008	Kurdistan	28.4	1505	134	E.coli	0.09	Culture	N.R
Kameli, 2013	Torbat Haidaria		1250	125	Staphylococcus epidermidis and E.coli	0.10	Urinalysis and culture	≥100
Kasraeian, 2009	Fars	29.3	389	20	E.coli	0.05	Culture	10^5
Keshavarz, 2007	Tehran (Hazrate Zainab Hospital)		900	33	E.coli and Staphylococcus saprophyticus	0.04	Urinalysis and culture	10^5
Mardanian, 2004	Isfahan		2345	270	NR	0.12	Urinalysis and culture	N.R
Mobasheri, 2000	Golestan		900	33	E.coli	0.04	Culture	10^5
Mojahedi, 2002	Mashhad	24.1	240	42	E.coli	0.18	Urinalysis and culture	10^4
Motaghi, 2012	Mashhad		150	16	E.coli	0.11	Urinalysis and culture	N.R
Namazi, 2012	Gilan	27.48	710	150	NR	0.21	Culture	10^5
Rahimkhani, 2012	Tehran(Emam Khomeini Hospital)		86	25	NR	0.29	Culture	10^5
Rahmanian, 2014	Semnan		160	9	NR	0.06	Culture	10^5
Shirazi, 2007	Hamadan		337	38	E.coli	0.11	Culture	10^5
Zarganjfard, 2000	Markazi		1736	110	E.coli	0.06	Urinalysis and culture	N.R

NR: not reported; NB: number of bacteria in 1 ml urine, NR: Not Report

### Heterogeneity

There was high heterogeneity among the results of included studies. Therefore, the Chi2 test was highly significant (*P*<0.001) and the I2 statistic was 98.8%. In order to reduce the heterogeneity, we performed subgroup analysis based on the quality of included studies, date of study conduction, and geographical region. Heterogeneity in low quality studies was 89.0% and studies that were conducted in the north of Iran was 99.0% (Table 2). Nonetheless, the heterogeneity in all subgroups was considerable.

**Table 2:** Subgroup analysis of the prevalence of ASB by quality of included studies, geographic regions, and the date of study conduction using Chi2 test for heterogeneity.

	Prevalence	95% CI	p-value	I2 (%)
**Quality of included studies**				
High	0.06	0.02, 0.09	-	-
Intermediate	0.14	0.08, 0.21	0.001	99.3
Low	0.10	0.08, 0.13	0.001	89.0
**Geographic region of Iran**				
North	0.13	0.09, 0.18	0.001	99.0
South	0.11	0.05, 0.16	0.001	95.1
**Year of study conduction**				
1996–2005	0.14	0.06, 0.22	0.001	99.5
2005–2013	0.13	0.09, 0.17	0.001	94.6

### Prevalence of asymptomatic bacteriuria

**Fig 2:**
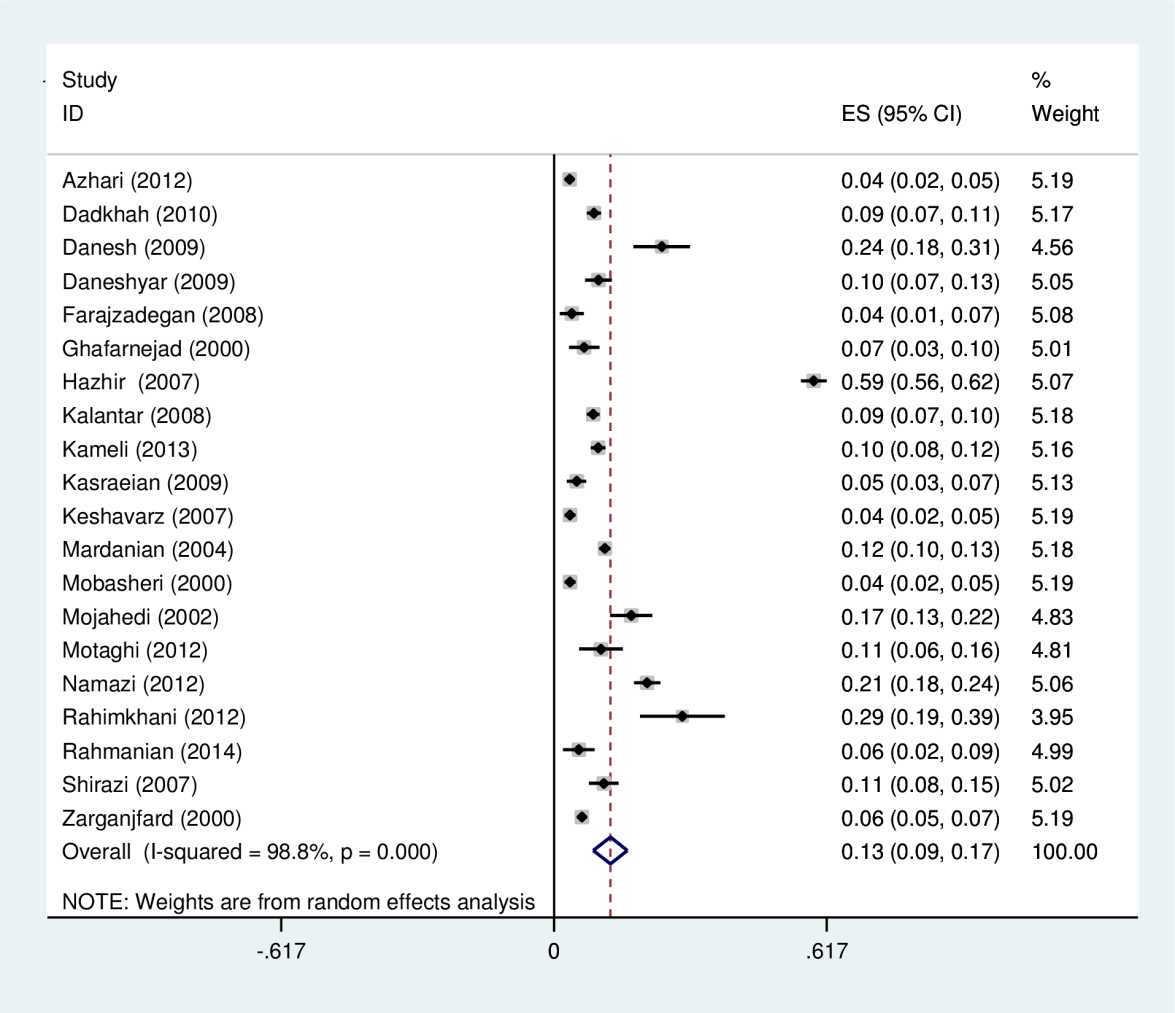
A forest plot for the prevalence of asymptomatic bacteriuria in pregnant women in Iran.

The overall prevalence of ASB among pregnant women was 0.13 (95% CI: 0.09, 0.17) (Fig 2). The prevalence of ASB in the northern and southern regions of Iran was 0.13 (95% CI: 0.09, 0.18) and 0.11 (95% CI: 0.05, 0.16), respectively. According to the quality based on STROBE checklist, the included studies were divided into three categories: 1 study (5%) had high quality, 12 studies (60%) had intermediate quality, and 7 studies (35%) had low quality. The prevalence of ASB in intermediate quality studies (0.14; 95% CI: 0.08, 0.21) was higher than studies with low quality (0.10; 95% CI: 0.08, 0.13). According to date of study conduction, included studies were divided into two groups: studies conducted from 1996 to 2005 (40%) and studies conducted from 2006 to 2013 (60%). Prevalence of ASB was lower into the recent studies (Table 2).

The type of detected bacteria in 8 (40%) of the included studies was unknown. In 9 (45%) of studies, the detected bacteria in pregnant women were Escherichia coli and in 2 (10%) of the included studies it was both E. coli and Staphylococcus. In1 (5%) study, S. epidermis were reported.

Four studies reported the antibiotic resistance among pregnant women with ASB in Iran. In Kameli study [10] the lowest percent of resistance was related to Amikacin (5%) and the highest percent was related to *sefixime*, amoxicillin and cotrimoxazole with 40%, 45% and 51% respectively. In Kalantar study [23] E.coli was resistant to ampicillin, cotrimoxazole, and nitrofurantoin with 89%, 70% and 20% respectively. In Keshavars study the lowest resistance of E.coli was related to gentamicin (7%) and the highest resistance was related to amoxicillin and oxacilin with 64%. In Shirazi et al., [34] the lowest resistance was related to ceftriaxone and ciprofloxacin with 3.8% and 7.7% respectively and the highest resistance was related to amoxicillin with 73.1%.

## Discussion

The results of this meta-analysis showed 13% of pregnant women in Iran had ASB. Our results indicated the difference in prevalence of ASB according to the date of study conduction in Iran was not considerable. Prevalence in recent studies (13%) was slightly lower than older studies (14%). In addition, the prevalence of ASB in southern regions was lower than northern regions. However, the difference was not substantial (11vs.13%).

According to the results of this meta-analysis, the prevalence of ASB among pregnant women is considerable in Iran. Some factors such as increase of age, sexual activity, history of urinary tract infection before pregnancy, socio economic status, several pregnancies and lack of personal hygiene increase the risk of ASB in pregnancy [35, 36].

There is a strong association between ASB and low birth weight in pregnant women [37]. In addition, ASB has other complications such as premature delivery [5]. Therefore, a proportion of low birth weight and premature delivery may be attributed to ASB among pregnant women in Iran. However, it seems that the design of studies, in order to determine the attributable fraction of ASB, is necessary to study complications of pregnant women in Iran.

The antibiotics resistance to some antibiotics such as ampicillin, cotrimoxazole amoxicillin, oxacilin and nitrofurantoin is high in Iran [10, 14, 23, 34]. On the other hand, a recently completed trial in the Netherlands has questioned the screen and treat approach to ASB in pregnant women [38]. They reported no increased risk of preterm birth in women with ASB. They also observed that while untreated or placebo treated women with asymptomatic bacteriuria had a 3.9 fold higher risk of pyelonephritis compared to asymptomatic bacteriuria negative women, the overall risk of pyelonephritis was low: ASB positive women developed pyelonephritis in five [2·4%] of 208 cases, compared with 24 [0·6%] of 4035 ASB negative. This is significant for Iran due to the high rates of antibiotic resistance but also because of the possible adverse effects of antibiotics on the neonate [38].

Prevalence of ASB in pregnant women in Asian countries such as Pakistan, Bangladesh, and India was reported to be 6–10.2% [11, 39, 40]. Prevalence of ASB in these mentioned studies was lower than our meta-analysis. However, the comparison between the results of our meta-analysis with cross-sectional studies may not be correct because the prevalence of ASB in the different regions of each country varies. In the addition, the prevalence of ASB in Iran varies from 4–29% [20, 32].

There was considerable heterogeneity (large I2 and a small *p*-value of the Chi2 test) among the results of included studies. Included studies were conducted in different regions of Iran. This difference may be a source of heterogeneity. However, the interpretation of a Chi2 test for heterogeneity should be taken with caution as the Chi2 test has limited capability when the sample size is small. On the other hand, the effectiveness of this test is high in identifying a small heterogeneity that might not be practically important [41]. In subgroups, based on the date of study conduction, geographical regions, and quality of included studies, the heterogeneity was high. Nonetheless, if the results of the meta-analysis are to be used as a guide for health decision-making, the meta-analysis of the heterogeneous results of studies is possible [42].

There were some limitations in this meta-analysis. First, the type of detected bacteria in eight studies was unknown. Therefore, we cannot determine the prevalent bacteria in pregnant women with ASB. A second limitation is the quality of included studies as only one study (5%) had high quality and 35% of them had low quality. This may increase the possibility of information bias.

## Conclusion

The results of this meta-analysis indicated the prevalence of ASB among Iranian pregnant women is considerable. Due to the complications of ASB for pregnant women and their children, preventative planning and control of ASB among pregnant women in Iran are necessary.

## Supporting Information

S1 File Reason of exclusion. (DOCX)

S2 File PRISMA Checklist. (DOC)
