# Correction: The Prevalence of Asymptomatic Bacteriuria in Iranian Pregnant Women: A Systematic Review and Meta-Analysis

**DOI:** 10.1371/journal.pone.0165114

**Published:** 2016-10-18

**Authors:** Mahin Ghafari, Vali Baigi, Zahra Cheraghi, Amin Doosti-Irani

Fig 1 is incorrect. Records excluded (n = 3342) is wrong. The records excluded should be (n = 3442). The authors have provided a corrected version here.

**Fig 1:**
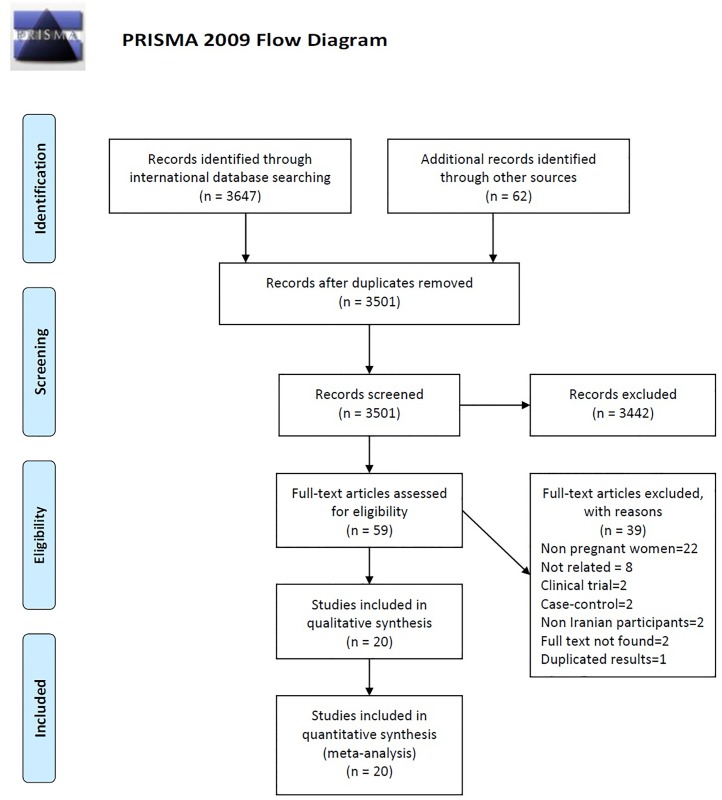
A flow chart depicting the stages of retrieving articles and checking eligibility criteria for meta-analysis. *From*: Moher D, Liberati A, Tetzlaff J, Altman DG, The PRISMA Group (2009). *P*referred *R*eporting *I*tems for Systematic Reviews and *M*eta-*A*nalyses: The PRISMA Statement. PLoS Med 6(7): e1000097. doi:10.1371/journal.pmed1000097. **For more information, visit** www.prisma-statement.org.
