# Supplementary material for: The Prevalence of Asymptomatic Bacteriuria in Iranian Pregnant Women: A Systematic Review and Meta-Analysis
Source: PLoS One. 2016 Jun 23;11(6):e0158031. doi: 10.1371/journal.pone.0158031 (PMC4919037; doi:10.1371/journal.pone.0158031)
Supplement: S1 File — (DOCX) [file pone.0158031.s001.docx]

Reasons of excluded studies from meta-analysis

| ID | Author | Date | Reason of exclusion |
| --- | --- | --- | --- |
| 1 | Ali isapour | 2015 | Participants were diabetic patients |
| 2 | Azarkish | 2003 | This study was a Randomized clinical trial |
| 3 | Gholamhossein Ettehad | 2006 | Participants were students |
| 4 | Seyed Mohammad Kazem Aghamir | 2010 | This study was a Randomized clinical trial |
| 5 | Khalifa Al Benwan | 2010 | This study was not related to the aim of our study |
| 6 | Agersew Alemu | 2012 | This study was not related to the aim of our study |
| 7 | Zohreh Aminzadeh | 2008 | This study was not related to the aim of our study |
| 8 | Anoush Azarfar | 2015 | This study was not related to the aim of our study |
| 9 | Mohammad Ali Boroumand | 2005 | Participants were no pregnant diabetic patients |
| 10 | Mohammad Ali Boroumand | 2006 | Participants were no pregnant diabetic patients |
| 11 | Anil Chander | 2013 | Study setting is Nepal |
| 12 | M. Gharouni | 2006 | Participants were not pregnant women |
| 13 | Behrouz Ghazimoghaddam | 2011 | This study was note related to the aim of our study |
| 14 | Sedigheh Hantush Zadeh | 2013 | This study was a case-control study |
| 15 | Nabi Jomezadeh | 2011 | Participants were children |
| 16 | M.Nasrolahei | 2013 | This study was not related to the aim of our study |
| 17 | Mohammad Nassaji | 2011 | Participants were not pregnant women |
| 18 | Yakubu Boyi Ngwai | 2012 | Study setting is Nigeria |
| 19 | G. Pouladfar | 2015 | This study was not related to the aim of our study |
| 20 | Gholamreza Pourmand | 2010 | Participants were patients who underwent prostatectomy |
| 21 | Mohammad Rahbar | 2012 | Participants were not pregnant women |
| 22 | Sepehr Salem | 2010 | Participants were not pregnant women |
| 23 | Farhad Sarrafzadeh | 2013 | Participants were not pregnant women |
| 24 | Ahmad Shajari | 2009 | Participants were school children |
| 25 | Nasrin Shayanfar | 2012 | Participants were not pregnant women |
| 26 | Ahmad Farajzadeh | 2011 | Participants were not pregnant women |
| 27 | Sofei-Majidpur | 2008 | Participants were not pregnant women |
| 28 | Behnam Zamanzad | 2007 | Participants were not pregnant women |
| 29 | Mohammad Ali Boroumand | 2004 | Participants were no pregnant diabetic women |
| 30 | Zahra Haji Amini | 2009 | Participants were not pregnant women |
| 31 | Fatemeh Dalaki | 2006 | Participants were not pregnant women |
| 32 | Narges Barghei | 2004 | This study was a case-control study |
| 33 | Masoud Sharifi | 2000 | Participants were not pregnant women |
| 34 | Masomeh Ghafarzadeh | 2001 | Full text not found |
| 35 | Motaghi M | 2010 | This study was not related to the aim of our study |
| 36 | Rezvan Moniri | 2009 | Participants were not pregnants women |
| 37 | Marzye Moosavi yazd | 2010 | Full text not found |
| 38 | Gholamhossein Ettehad | 2007 | Participants were students |
| 39 | Mohammad Ali Boroumand | 2002 | Duplicated |
